# Activation of the Monocyte/Macrophage System and Abnormal Blood Levels of Lymphocyte Subpopulations in Individuals with Autism Spectrum Disorder: A Systematic Review and Meta-Analysis

**DOI:** 10.3390/ijms232214329

**Published:** 2022-11-18

**Authors:** Gara Arteaga-Henríquez, Jorge Lugo-Marín, Laura Gisbert, Imanol Setién-Ramos, Mónica Martínez-Gallo, Ricardo Pujol-Borrell, Josep Antoni Ramos-Quiroga

**Affiliations:** 1 Department of Psychiatry, Hospital Universitari Vall d’Hebron (HUVH), 08035 Barcelona, Catalonia, Spain; 2 Biomedical Network Research Centre on Mental Health (CIBERSAM), 28029 Madrid, Madrid, Spain; 3 Psychiatric Genetics Unit, Group of Psychiatry, Mental Health and Addiction, Vall d’Hebron Insitute (VHIR), Universitat Autònoma de Barcelona, 08035 Barcelona, Catalonia, Spain; 4 Department of Psychiatry and Legal Medicine, Universitat Autònoma de Barcelona, 08035 Barcelona, Catalonia, Spain; 5 Department of Immunolgy, Hospital Universitari Vall d’Hebron (HUVH), 08035 Barcelona, Catalonia, Spain; 6 Diagnostic Immunology Research Group, Vall d’Hebron Research Institute (VHIR), 08035 Barcelona, Catalonia, Spain; 7 Department of Cell Biology, Physiology and Immunology, Universitat Autònoma de Barcelona (UAB), 08193 Barcelona, Catalonia, Spain

**Keywords:** ASD, monocytes, lymphocytes, inflammation, immune

## Abstract

Autism spectrum disorder (ASD) is a neurodevelopmental condition with a so far unknown etiology. Increasing evidence suggests that a state of systemic low-grade inflammation may be involved in the pathophysiology of this condition. However, studies investigating peripheral blood levels of immune cells, and/or of immune cell activation markers such as neopterin are lacking and have provided mixed findings. We performed a systematic review and meta-analysis of studies comparing total and differential white blood cell (WBC) counts, blood levels of lymphocyte subpopulations and of neopterin between individuals with ASD and typically developing (TD) controls (PROSPERO registration number: CRD CRD42019146472). Online searches covered publications from 1 January 1994 until 1 March 2022. Out of 1170 publication records identified, 25 studies were finally included. Random-effects meta-analyses were carried out, and sensitivity analyses were performed to control for potential moderators. *Results:* Individuals with ASD showed a significantly higher WBC count (k = 10, *g* = 0.29, *p* = 0.001, *I*^2^ = 34%), significantly higher levels of neutrophils (k = 6, *g* = 0.29, *p* = 0.005, *I*^2^ = 31%), monocytes (k = 11, *g* = 0.35, *p* < 0.001, *I*^2^ = 54%), NK cells (k = 7, *g* = 0.36, *p* = 0.037, *I*^2^ = 67%), Tc cells (k = 4, *g* = 0.73, *p* = 0.021, *I*^2^ = 82%), and a significantly lower Th/Tc cells ratio (k = 3, *g* = −0.42, *p* = 0.008, *I*^2^ = 0%), compared to TD controls. Subjects with ASD were also characterized by a significantly higher neutrophil-to-lymphocyte ratio (NLR) (k = 4, g = 0.69, *p* = 0.040, *I*^2^ = 90%), and significantly higher neopterin levels (k = 3, g = 1.16, *p* = 0.001, *I*^2^ = 97%) compared to TD controls. No significant differences were found with respect to the levels of lymphocytes, B cells, Th cells, Treg cells, and Th17 cells. Sensitivity analysis suggested that the findings for monocyte and neutrophil levels were robust, and independent of other factors, such as medication status, diagnostic criteria applied, and/or the difference in age or sex between subjects with ASD and TD controls. Taken together, our findings suggest the existence of a chronically (and systemically) activated inflammatory response system in, at least, a subgroup of individuals with ASD. This might have not only diagnostic, but also, therapeutic implications. However, larger longitudinal studies including more homogeneous samples and laboratory assessment methods and recording potential confounding factors such as body mass index, or the presence of comorbid psychiatric and/or medical conditions are urgently needed to confirm the findings.

## 1. Introduction

Autism spectrum disorder (ASD) is considered a neurodevelopmental condition characterized by persistent deficits in social communication and by restrictive, repetitive and/or stereotyped behaviors [1]. With a current global prevalence of about 1%, ASD is associated with a high community and individual burden. Furthermore, individuals with ASD are at a high risk of self-harm and committing suicide [2]. Despite this, the etiology of ASD remains not yet fully understood [3], and in the absence of clear biological markers, the diagnosis if this condition is still clinical and treatment options insufficient. Increasing evidence suggests the existence of a dysregulated immune system as a key factor involved in the pathogenesis of this condition. Maternal immune activation (MIA) is known to increase the risk for ASD in the offspring [4], genome-wide association studies have revealed that variations in genes encoding proteins involved in the inflammatory response (e.g., *HLA*) might be associated with ASD [5,6], and individuals with ASD often display abnormal immune responses [7]. Moreover, increasing evidence has reported that subjects with ASD are at a high risk of suffering from immune-mediated comorbidities, such as psoriasis [8]. However, and despite the great amount of literature linking ASD with immune system abnormalities, no consistent and specific immunological dysfunction has emerged so far. A variety of mechanisms have been suggested. One mechanism involves the chronic activation of microglia, monocytes, and/or T lymphocytes, impacting on brain development and function. This proposed mechanism is consistent with the cytokine profile described in ASD, with individuals with ASD showing increased levels of pro-inflammatory cytokines (i.e., interleukin (IL)-1B, IL-6, interferon gamma (IFN-y)) [9,10], and decreased levels of anti-inflammatory cytokines (i.e., transforming growth factor (TFG)-β, IL-10) [11] in their blood and/or cerebrospinal fluid (CSF). Cytokines comprise a group of more than 100 peptides primarily produced by activated cells of the immune system. However, it should be emphasized these compounds are also produced by other cell types, such as adipocytes [12], turning them into molecules of a rather unspecific nature. Therefore, exploring abnormalities in the levels of immune cells or in the levels of other (more specific) immune cell activation markers, such as neopterin (which is almost exclusively produced by macrophages/monocytes and/or T cells) [13], would be of interest. The literature on both immune cell and/or neopterin levels is sparse and has provided mixed findings. The aim of this study was to investigate the existence of abnormalities in peripheral blood levels of immune cells and of neopterin between individuals with ASD and typically developing (TD) controls by using a meta-analytic approach. 

## 2. Methods

This systematic review and meta-analysis were undertaken and reported according to the Preferred Reporting Items for Systematic Reviews and Meta-analyses (PRISMA) guidelines [14]. The PRISMA checklist is shown in Supplementary Table S1. The study protocol was registered at the International Prospective Register of Systematic Reviews (PROSPERO) (Protocol ID number: CRD42019146472).

### 2.1. Study Selection

#### 2.1.1. Eligibility Criteria

In-and exclusion criteria were discussed and approved by all authors. The following inclusion criteria were applied: (1) English-, Spanish, German, and/or French-written observational studies showing comparative data on total and differential white blood cell (WBC) counts, or on blood levels of lymphocyte subpopulations or neopterin between individuals with ASD and TD healthy controls, (2) ASD diagnosis according to DSM-IV, DSM-IV-TR, DSM-5 and/or ICD-10 criteria, (3) necessary data available (i.e., mean, median, standard deviation (SD), standard error (SE), range or interquartile range (IQR) for the levels of immune/inflammatory markers, as well as the numbers of individuals with ASD and TD healthy controls). Excluded were studies meeting at least one of the following criteria: (1) non-original studies (e.g., reviews, commentaries, editorials, book chapters), (2) non peer-reviewed studies (i.e., conference abstracts), patents, safety and/or non-comparative studies (i.e., case reports, case series), (3) animal model studies, (4) studies focusing on certain tissue/cerebrospinal fluid instead of peripheral blood, (5) studies assessing genetic polymorphisms, (6) targeted sampling strategies (i.e., specifically selected patients with any comorbidity, or under a specific medication), (7) individuals with Rett’s syndrome, (8) significant study overlap (i.e., the same patient and/or control group in multiple publications), and (9) studies using siblings as controls. Grey literature was not included. 

#### 2.1.2. Search Strategy

**Figure 1:**
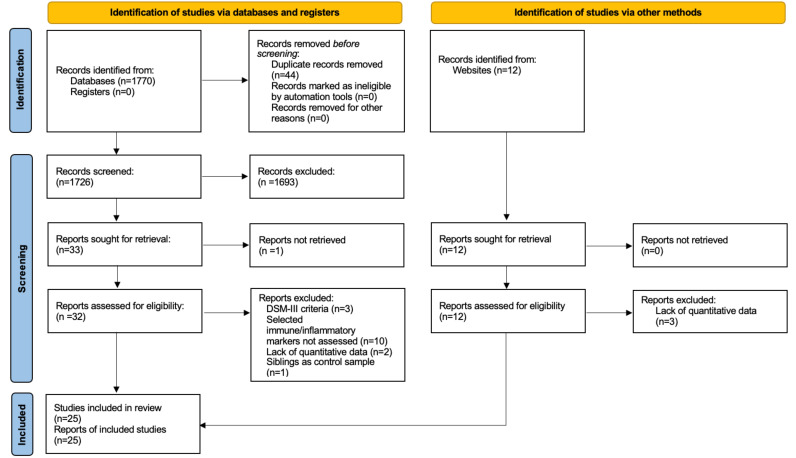
Flow chart of study selection process.

PubMed, SCOPUS, and World of Knowledge electronic databases were independently searched by two authors (GA-H, JL-M) for relevant articles published between 1 January 1994 and 1 March 2022. The following search syntax (which was first discussed and approved by all authors) was used for PubMed database search, and adapted according to the different database index terms: (autism OR ASD OR autistic* OR asperger*) AND (immun*[Title/Abstract] OR inflamm*[Title/Abstract] OR T cells[Title/Abstract] OR monocytes[Title/Abstract] OR neutrophils [Title/Abstract] OR NLR [Title/Abstract] OR NK cells[Title/Abstract] OR natural killer[Title/Abstract] OR T helper[Title/Abstract] OR Th[Title/Abstract] OR T cytotoxic[Title/Abstract] OR Tc[Title/Abstract] OR T regulatory[Title/Abstract] OR Treg[Title/Abstract] OR Tregs[Title/Abstract] OR neopterin[Title/Abstract]). Title and abstracts of all electronic articles retrieved were independently screened by two authors (GA-H, JL-M). Reference lists of included studies and relevant topic-related reviews were manually screened to retrieve any additional reports meeting criteria. Articles presumed to meet the inclusion criteria were retrieved as full-texts, and independently examined by the same authors, reaching a preliminary list of included studies. In case of disagreement, a consensus including all authors was reached. A final list of included articles was then reached. A flow-chart summarizing the study selection process is presented in Figure 1. 

### 2.2. Data Extraction

One author (GA-H) extracted all data, the method used to extract the data was independently verified by two other authors (LG, ISR) and data were re-checked for accuracy by a third author (JL-M). Data extracted included: name of first author, date of publication, country of origin, population description (i.e., index and control sample size, age, sex, body mass index (BMI), medication (yes/no/not given), diagnostic criteria to establish a diagnosis of ASD, total and differential WBC and blood levels of lymphocyte subpopulations and/or neopterin. Whenever possible, data were retrieved as mean and standard deviation (SD). Conversion methods were used to calculate mean and SD in case of data provided as median, range, interquartile range (IQR), and standard error (SE). The median was included as an approximation of the mean (Higgins and Green, 2011); the SD was calculated using the following formulas: SD = IQR/1.35; SD = SE × √N, or SD = maximum-minimum/4 [15,16].Correspondence and first authors of possibly relevant articles, which were either not available as full-text, or could qualify, but information on eligibility was judged as insufficient (i.e., lack of quantitative data), were contacted by email (two times) by two authors (GA-H, JL-M). 

### 2.3. Quality Assessment

Two researchers (GA-H, LG) independently evaluated the quality of included studies according to the Newcastle-Ottawa Scale (NOS) [17]. The NOS was developed to evaluate three categories of quality: (1) the selection of study groups (maximum score: 4 stars), (2) the comparability of study groups (maximum score: 2 stars), and (3) the ascertainment of exposure or outcome of interest (maximum score: 3 stars). The NOS ranges from 0 to 9 stars (the higher the score, the better the quality of the study). Disagreements regarding the quality of inclusion studies were resolved through discussion with a third author (IS-R). 

### 2.4. Data Analysis

Differences in blood levels of immune/inflammatory markers between individuals with ASD and TD controls were tested as main outcome measures. Meta-analyses were conducted when two or more studies were included using a similar modality of biomarker assessment. When multiple studies investigated the same sample, analysis included only the study with the largest sample size. Effect size (ES) estimates were based on Hedge’s g and their 95% CI for every individual marker, considering Hedge’s values from 0.2 to 0.49 small, values from 0.5 to 0.79 moderate, and greater than 0.8, large. Based on the expected between-study differences in sample size and/or recruitment procedures and methods used to determine the levels of assessed markers, random-effects models were used [18]. A positive ES implied that higher levels of the specified immune/inflammatory marker were reported in the ASD group compared to the TD control group. Between-study heterogeneity was assessed by applying the *I*^2^ statistic; heterogeneity was considered moderate when *I*^2^ ranged between 50% and 75%, and high when *I*^2^ was greater than 75% [19]. Publication bias was assessed by visual inspection of funnel plots asymmetry, and by the Egger’s test, if the levels of specific immune/inflammatory markers were determined by at least 10 studies [20]. The Orwin’s fail-safe N was also applied, generating the number of missing or unpublished studies required to move ES to irrelevant values (i.e., Hedge’s g under 0.2). When the funnel plot or test statistics suggested publication bias, the Duval and Tweedie trim-and-fill method was applied, in order to estimate and correct ES for publication bias. In addition, the leave-one-out sensitivity analysis was also performed with the aim of investigating if any single study accounted for between-study heterogeneity. This consists of iteratively repeating the meta-analysis excluding one study at a time to establish whether the results are replicable. Subgroup sensitivity analyses including data from medication-naïve samples, and from samples diagnosed according to DSM-IV/ICD-10 and/or DSM-5 criteria were also performed. In addition, meta-regression analyses were carried out in case of continuous moderators that were assessed by at least six studies [21]. Data analysis was performed using the Comprehensive Meta-analysis software v.3.1.1. *p*-values were considered statistically significant at the α = 0.05 level. 

## 3. Results

### 3.1. General Characteristics of Eligible Studies

Online searches identified 1770 publication records; 25 studies [22,23,24,25,26,27,28,29,30,31,32,33,34,35,36,37,38,39,40,41,42,43,44,45,46] were finally included in the meta-analysis according to the pre-specified in-and exclusion criteria (Figure 1). General characteristics of studies included are presented in Table 1. Children and adolescents were recruited by the vast majority of studies, except for one that was based on adult participants [34], and for another one, where a non-specified number of adults was included [35]; age range: 3–22 years. All studies included were age- and sex-matched, i.e., there were no significant differences between individuals with ASD and TD controls with respect to age and sex. ASD was diagnosed according to DSM-IV/ICD-10 criteria in fifteen publications [22,23,24,25,26,27,28,29,30,32,36,37,38,39,43], and to DSM-5, in nine publications [31,33,34,35,40,41,44,45,46]. Two register-based studies were included [38,39]. Medication status was not specified in twelve publications [23,24,26,27,28,30,34,36,37,40,41,42]. Individuals with ASD were medication-naïve in twelve studies [22,25,29,31,32,33,35,38,39,43,45,46]; in the remaining study [44], approximately 56% of subjects with ASD were under medication. The NOS score ranged between 2 and 8 (Supplementary Table S2). The following immune/inflammatory parameters were determined in the eligible studies: WBC count, blood levels of neutrophils, monocytes, lymphocytes, B cells, NK cells, T cells, T cytotoxic (Tc) cells, T helper (Th) cells, Th17 cells, T regulatory (Treg) cells, neopterin, the Th/Tc cells ratio, the neutrophil-to-lymphocyte ratio (NLR) (Supplementary Table S3). 

### 3.2. WBC

The WBC count was assessed by ten studies [27,29,31,32,37,38,39,40,41,44]. Overall, individuals with ASDs were characterized by a significantly higher WBC count compared to TD controls (k = 10, Hedge’s *g* = 0.29, 95% CI: 0.13–0.45, *p* = 0.001) (Table 2). Heterogeneity among studies was low (*I*^2^ = 34%) (Table 2). After removing single studies in the sensitivity analysis, the difference in the WBC remained significant (Supplementary Table S4). Results of the Egger’s test were not significant, indicating no evidence of publication bias (Table 2). Sensitivity subgroup meta-analyses remained significant when the analysis was limited to studies based on individuals with ASD diagnosed according to DSM-IV/ICD-10 criteria (k = 6, *g* = 0.209, 95% CI: 0.03–0.39, *p* = 0.023, *I*^2^ = 25%) or to DSM-5 criteria (k = 4, *g* = 0.44, 95% CI: 0.15–0.72, *p* = 0.003, *I*^2^ = 30%). On the contrary, sensitivity subgroup meta-analyses appeared not significant when the analysis was limited to studies based on medication-naïve individuals with ASD (k = 5, *g* = 0.14, 95% CI: −0.06–0.35, *p* = 0.167, *I*^2^ = 17%). Meta-regression analyses showed that the ES for the difference in the WBC count between individuals with ASD and TD were not significantly associated with moderators age, sex, and/or year of publication (Supplementary Table S5).

### 3.3. Neutrophils

Blood levels of neutrophils were assessed by six studies [27,31,38,39,44,46]. Overall, individuals with ASD were characterized by higher blood levels of neutrophils compared to TD controls (k = 6, *g* = 0.29, 95% CI: 0.08–0.49, *p* = 0.005) (Table 2). Heterogeneity among studies was low (*I*^2^ = 31%) (Table 2). After removing any of the single studies in the sensitivity analysis, the difference in the monocyte levels remained significant (Supplementary Table S4). Meta-regression analyses showed that the ES for the difference in neutrophil blood levels between individuals with ASD and TD controls were, again, not significantly associated with moderators age, sex, and/or year of publication (Supplementary Table S5). 

### 3.4. Monocytes

**Figure 2:**
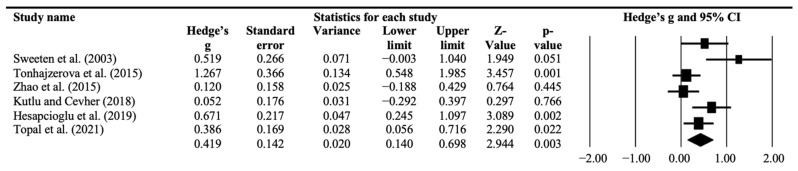
Forest plot of cross-sectional comparisons of blood monocyte levels between medication-naïve individuals with ASD and TD controls. Study name [22,31,32,38,39,46].

Blood monocyte levels were assessed by 11 studies [22,27,28,31,32,34,37,38,39,44,46]. Overall, individuals with ASD were characterized by higher blood levels of monocytes compared to TD controls (k = 11, *g* = 0.35, 95% CI: 0.17–0.54, *p* < 0.001) (Table 2). Heterogeneity among studies was moderate (*I*^2^ = 54%) (Table 2). After removing any of the single studies in the sensitivity analysis, the difference in the monocyte levels remained significant (Supplementary Table S4). Results of the Egger’s test appeared not significant, indicating no evidence of publication bias (Table 1). Sensitivity subgroup meta-analyses including only studies with medication-naïve individuals with ASD confirmed the direction, magnitude and significance of the associations for monocyte levels (k = 6, *g* = 0.42, 95% CI: 0.14–0.69, *p* = 0.003, *I*^2^ = 64%) (Figure 2). The difference between groups remained significant too, when the analysis was limited to studies based on participants with an ASD diagnosed according to DSM-IV/ICD-10 criteria (k = 7, *g* = 0.42, 95% CI: 0.140–0.698, *p* = 0.003, *I*^2^ = 37%), or to DSM-5 criteria (k = 4, *g* = 0.55, 95% CI: 0.14–0.95, *p* = 0.008, *I*^2^ = 68%). Meta-regression analyses showed that the ES for the difference in monocyte levels between individuals with ASD and TD was not significantly associated with age, sex, and/or year of publication (Supplementary Table S5).

### 3.5. Lymphocytes

Blood lymphocyte levels were assessed by six studies [31,32,37,38,39,46]. Overall, no significant differences in the levels of lymphocytes were found between individuals with ASD and TD controls (k = 6, *g* = −0.055, 95% CI: −0.229–0.119, *p* = 0.533, *I*^2^ = 24%) (Table 1). After removing any of the single studies in the sensitivity analysis, the difference in the monocyte levels remained not significant (Supplementary Table S4). The difference between groups remained not significant when the analysis was limited to studies based on medication-naïve participants (k = 5, *g* = −0.044, 95% CI: −0.264–0.176, *p* = 0.695, *I*^2^ = 39%), or to studies based on participants with an ASD diagnosed according to DSM-IV/ICD-10 criteria (k = 4, *g* = −0.126, 95% CI: −0.317–0.064, *p* = 0.194, *I*^2^ = 17%), or to DSM-5 criteria (k = 2, *g* = 0.131, 95% CI: −0.162–0.425, *p* = 0.381, *I*^2^ = 0%). Meta-regression analyses showed that the ES for the difference in the monocyte levels between individuals with ASD and TD was not significantly associated with moderators age, sex, and/or year of publication (Supplementary Table S5). 

### 3.6. B Cells

The levels of B cells were assessed by five studies [25,27,28,29,34]. Overall, no statistically significant differences in the levels of B cells were found between individuals with ASD and TD controls (k = 5, *g* = −0.09, 95% CI: −0.58–0.40, *p* = 0.722, *I*^2^ = 80%) (Table 1). After removing single studies in the sensitivity analysis, the difference in B cell levels remained not significant (Supplementary Table S4). Sensitivity subgroup meta-analyses appeared significant when the analysis was limited to studies based on medication-naïve individuals with ASD (k = 2, *g* = −0.56, 95% CI: −0.98–−0.14, *p* = 0.009, *I*^2^ = 0%). Sensitivity subgroup meta-analyses based on studies where individuals with ASD were diagnosed according to DSM-IV/ICD-10 criteria confirmed the direction, magnitude and significance of the associations for B cell levels (k = 4, *g* = −0.25, 95% CI: −0.79–0.29, *p* = 0.370, *I*^2^ = 78%). Due to the low number of studies, subgroup analyses based on participants with ASD diagnosed according to DSM-5 criteria were not performed, and potential moderators were not tested. 

### 3.7. NK Cells

The levels of NK cells were assessed by seven studies [23,25,27,29,34,35,45]. Overall, significantly higher NK cell levels were found in individuals with ASD compared to TD controls (k = 7, *g* = 0.36, 95% CI: 0.02–0.71, *p* = 0.037) (Table 1). Heterogeneity among studies was moderate (*I*^2^ = 67%) (Table 1). After removing any of the single studies in the sensitivity analysis [23,27,29,35], the difference in NK cells appeared not significant (Supplementary Table S4). Subgroup analysis revealed that the difference between groups appeared not significant when the analysis was limited to studies based on medication-naïve participants (k = 4, *g* = 0.36, 95% CI: −0.37–1.09, *p* = 0.336, *I*^2^ = 82%), on participants with ASD diagnosed according to DSM-IV/ICD-10 criteria (k = 3, *g* = 0.59, 95% CI: −0.06–1.23, *p* = 0.074, *I*^2^ = 77%), and/or participants with ASD diagnosed according to DSM-5 criteria (k = 3, *g* = 0.11, 95% CI: −0.36–0.58, *p* = 0.65, *I*^2^ = 65%). Meta-regression analyses demonstrated that between-group differences in age, sex, and year of publication were not significantly associated with ES estimates for NK cell levels (Supplementary Table S5).

### 3.8. T Cells

T cell levels were assessed by five studies [25,27,28,29,34]. Overall, no significant differences were found in the levels of T cells between individuals with ASD and TD controls (k = 5, *g* = 0.024, 95% CI: −0.367–0.414, *p* = 0.905, *I*^2^ = 68%) (Table 1). After removing single studies in the sensitivity analysis, the difference in T cell levels remained not significant (Supplementary Table S4). Subgroup analysis revealed that the difference between groups remained not significant when the analysis was limited to studies based on medication-naïve participants (k = 2, *g* = −0.14, 95% CI: −1.36–1.07, *p* = 0.817, *I*^2^ = 88%), and on participants with ASD diagnosed according to DSM-IV/ICD-10 (k = 4, *g* = 0.08, 95% CI: −0.41–0.57, *p* = 0.757, *I*^2^ = 73%). Due to the low number of studies, subgroup analysis based on participants with ASD diagnosed according to DSM-5 criteria were not performed, and potential moderators were not tested.

### 3.9. Tc Cells

The levels of Tc cells were assessed by four studies [25,27,29,34]. Overall, significantly higher levels of Tc cells were found in individuals with ASD compared to TD controls (k = 4, *g* = 0.726, 95% CI: 0.111–1.342, *p* = 0.021) (Table 1). Heterogeneity was, however, high (82%). After removing any of the single studies [25,29,34] in the sensitivity analysis, the difference in the levels of Tc cells appeared to be not significant (Supplementary Table S4). Subgroup analysis revealed that the difference between groups appeared not significant when the analysis was limited to studies based on medication-naïve participants (k = 2, *g* = 1.04, 95% CI: −0.05–2.13, *p* = 0.062, *I*^2^ = 83%), and on participants with ASD diagnosed according to DSM-IV/ICD-10 (k = 3, *g* = 0.69, 95% CI: −0.18–1.57, *p* = 0.118, *I*^2^ = 87%). Due to the low number of studies, subgroup analysis based on participants with an ASD diagnosed according to DSM-5 criteria could not be performed; potential moderators were also not tested.

### 3.10. Th Cells

The levels of Th cells were assessed by five studies [25,27,29,34,36]. Overall, significant differences between individuals with ASD and TD controls in relation to their levels of Th cells were not found (k = 5, *g* = −0.35, 95% CI: −1.10–0.41, *p* = 0.370) (Table 1). Heterogeneity was high (92%). After removing single studies in the sensitivity analysis, the difference in Th cell levels remained not significant (Supplementary Table S4). Subgroup analysis revealed that the difference between groups remained not significant when the analysis was limited to studies based on medication-naïve participants (k = 2, *g* = −0.51, 95% CI: −2.74–1.72, *p* = 0.651, *I*^2^ = 96%), and on participants with ASD diagnosed according to DSM-IV/ICD-10 (k = 4, *g* = −0.47, 95% CI: −1.41–0.48, *p* = 0.332, *I*^2^ = 93%). Due to the low number of studies, subgroup analysis based on participants with ASD diagnosed according to DSM-5 criteria could not be performed, and potential moderators were not tested.

### 3.11. Th/Tc Cells Ratio

The Th/Tc cells ratio was assessed by three studies [25,29,34]. Overall, a significantly lower Th/Tc cells ratio was found in individuals with ASD when compared to TD controls (k = 3, *g* = −0.42, 95% CI: −0.73–−0.11, *p* = 0.008) (Table 1); heterogeneity among studies was negligible (*I*^2^ = 0%). Sensitivity analyses were not performed. 

### 3.12. Th17 Cells

The levels of Th17 cells were assessed by three studies [24,33,40]. Overall, individuals with ASD were characterized by a trend of higher Th17 cell levels compared to TD controls (k = 3, *g* = 1.75, 95% CI: −0.25–4.37, *p* = 0.081) (Table 1). Heterogeneity was high (*I*^2^ = 97%) (Table 1); after removing one study [24] in the sensitivity analysis, the difference in Th17 cell levels appeared to be significant (Supplementary Table S4). The difference also appeared significant when subgroup analysis on individuals with ASD who were diagnosed according to DSM-5 criteria were performed (k = 2, *g* = 3.07, 95% CI: 0.13–6.01, *p* = 0.041, *I*^2^ = 97%). Individuals with ASD were diagnosed according to DSM-IV/ICD-10 criteria in only one study [24]; medication-status was specified only in one study [33]. Due to the low number of studies, potential moderators were not tested.

### 3.13. Treg Cells

The levels of Treg cells were assessed by seven studies [26,29,33,34,40,43,45]. Overall, a trend of significantly lower Treg cell levels was found in the blood of individuals with ASD compared to TD controls (k = 7, *g* = −1.30, 95% CI: −2.61–0.04, *p* = 0.051) (Table 1). Heterogeneity was, however, high (*I*^2^ = 97%) (Table 1). After removing one study [29] in the sensitivity analysis, the difference in Treg cell levels appeared to be significant (Supplementary Table S4). Subgroup analysis revealed that the difference between groups appeared significant when the analysis was limited to studies based on an ASD diagnosis according to DSM-5 criteria (k = 4, *g* = −2.05, 95% CI: −3.95–−0.15, *p* = 0.035, *I*^2^ = 97%). The difference remained not significant when the analysis was limited to studies based on medication-naïve participants (k = 4, *g* = −1.01, 95% CI: −3.13–1.12, *p* = 0.354, *I*^2^ = 97%), and on participants with an ASD diagnosis based on DSM-IV/ICD-10 criteria (k = 3, *g* = −0.31, 95% CI: −2.14–1.52, *p* = 0.741, *I*^2^ = 96%). Meta-regression analyses demonstrated that between-group differences in age, sex, and/or year of publication were not significantly associated with ES estimates for Treg cell levels (Supplementary Table S5). 

### 3.14. NLR

The NLR was assessed by four studies [38,39,42,46]. Overall, individuals with ASD were characterized by a higher NLR compared to TD controls (k = 4, *g* = 0.69, 95% CI: 0.03–1.34, *p* = 0.040) (Table 1). Heterogeneity among studies was high (*I*^2^ = 90%) (Table 1). After removing any of single studies [38,42,46] in the sensitivity analysis, the difference in the NLR appeared to be not significant (Supplementary Table S4). Subgroup analysis revealed that a trend of higher NLR was found in individuals with ASD compared to TD controls when the analysis was limited to studies based on medication-naïve participants [38,39,46] (k = 3, *g* = 0.16, 95% CI: −0.01–0.63, *p* = 0.057). Due to the low number of studies, potential moderators were not tested.

### 3.15. Neopterin

Blood neopterin levels were assessed by three studies [22,30,32] (Table 1). Overall, individuals with ASD were characterized by higher levels of neopterin when compared to TD controls (k = 3, *g* = 1.16, 95% CI: 0.62–1.69, *p* = 0.001) (Table 1). Heterogeneity among studies was moderate (*I*^2^ = 72%) (Table 1). After removing single studies in the sensitivity analysis, the difference in the monocyte levels remained significant (Supplementary Table S4). Due to the low number of studies, potential moderators were not tested. Sensitivity subgroup meta-analyses including only studies with medication-naïve individuals with ASD confirmed the direction, magnitude and significance of the associations for neopterin levels. In all included studies, ASDs were diagnosed according to DSM-IV criteria. Therefore, sensitivity subgroup meta-analyses based on the diagnostic criteria applied for diagnosing ASD were, in this case, not performed.

## 4. Discussion

To the best of our knowledge, this is the largest meta-analysis to date exploring differences in blood peripheral levels of immune cells and immune cell activation markers (i.e., neopterin) between individuals with ASD and TD controls. Overall, 25 studies were included. We found that subjects with ASD were characterized by a significantly higher WBC count, and by significantly higher neutrophil and monocyte blood levels, compared to TD controls. ES was low for all the above-mentioned immune markers. Heterogeneity was low for the WBC count and for neutrophil levels, and moderate for monocyte levels. Sensitivity analyses suggested that the findings related to neutrophil and monocyte levels were robust, and independent of other factors, such as medication status, diagnostic criteria applied, year of publication, and/or the differences between subjects with ASD and TD in relation to age and/or sex. The WBC consists of different cell types (i.e., neutrophils, eosinophils, basophils, monocytes, lymphocytes, plasmatic cells) and it is currently considered as a reliable measure of the overall immune system activity. In other words, a high WBC count (i.e., leukocytosis) suggests the existence of an activated response system (IRS) [47]. While the initial characteristic of acute inflammation is an increase in the levels of neutrophils, chronic inflammation is associated with an increase in the levels of mononuclear cells, including monocytes. Therefore, our findings suggest the existence of a chronic activated IRS in individuals with ASD. In line with this idea, an increased expression of several activation markers (e.g., CD96, HLA-DR) has been demonstrated in the circulating monocytes of subjects with ASD. In addition, higher levels of pro-inflammatory cytokines (e.g., interleukin (IL)-6; interferon gamma (IFN-γ)), and lower levels of anti-inflammatory cytokines (e.g., transforming growth factor (TGF)-β, IL-10) have been also found in the blood of individuals with ASD [9,10,11]. Interestingly, significantly higher blood neopterin levels were also found in individuals with ASD compared to TD controls. ES was large; heterogeneity was, however, high. Neopterin is primarily produced and secreted by activated macrophages and/or monocytes, and contrary to what happens with other compounds, such as cytokines, it is hardly produced by other cell types, apart from T cells. Therefore, it is considered as a very specific immune cell activation marker [48]. One of the main triggers for neopterin production is IFN-γ. As mentioned before, increased peripheral levels of IFN-γ have been repeatedly demonstrated in individuals with ASD [9,10]. Interestingly, IFN-γ is mainly released from activated T cells, such as Tc (CD8^+^) cells [49]. In addition, interestingly, our study showed that individuals with ASD were characterized by significantly increased levels of Tc cells, and by a significantly lower Th/Tc cells ratio, compared to TD controls. ES was moderate for Tc cells, and low for the Th/Tc cells ratio. Heterogeneity was high for Tc cells, and negligible for the Th/T cells ratio. Sensitivity analyses revealed that between-group differences in Tc cell levels were no longer significant after removing several single studies, indicating that the effect might depend on other factors, such as medication status and/or diagnostic criteria applied. However, in a recent study performed by DiStasio and colleagues [50], increased levels of Tc lymphocytes, and decreased levels of Th lymphocytes were found in the postmortem brains of a cohort of individuals with ASD [50], supporting our findings. Importantly, monocytosis has been repeatedly associated with high levels of activated microglia in the brain [51], suggesting that the state of chronic activation of the IRS (and in particular, of the monocyte/macrophage system) in individuals with ASD might be systemic (including the central nervous system) and not restricted to the periphery. In support of this idea, increasing evidence has reported an upregulation of pro-inflammatory genes and microglia activation markers in the brain of individuals with ASD [52,53]. In addition, increased levels of pro-inflammatory cytokines and/or chemokines have been also repeatedly reported in the brain and/or cerebrospinal fluid (CSF) of subjects with ASD across their lifespan. Our study also showed that individuals with ASD were characterized by significantly higher NK cell levels compared to TD controls. ES was small and heterogeneity moderate. NK cells have been classically considered as critical cells to contain viral infections. However, under inflammatory conditions, NK cells can migrate to the brain and dampen down microglial inflammatory activity, acting as a kind of regulatory cell [54]. Since accumulating research has however reported the existence of a reduced activity of these cells in individuals with ASD [55,56], we hypothesize that the increase in blood peripheral levels of NK cells might reflect a compensatory (but failed) mechanism aimed at dampening down systemic inflammation in ASD. 

Our findings might have diagnostic and therapeutic implications. A significant positive association between the NLR and/or blood levels of neopterin, and the severity of ASD (as assessed by the total CARS and/or GARS-2 scores) have been reported in individuals with ASD [32,39,46]. Other studies have reported and association between the levels of neutrophils and the severity of core symptoms, such as social interaction deficits, in individuals with ASD [46]. Interestingly, in a study performed in a sample of 12 children with ASD, an association between pre-treatment immune status and response to naltrexone was demonstrated, with low pretreatment percentages of Th cells, and high pretreatment percentages of Tc cells predicting non-response to this agent [57]. Potential advantages for the use of these parameters to diagnose and guide treatments include that they are rapidly available, inexpensive, easy to interpret, and could thus be routinely ordered [58]. 

Although encouraging, our findings should be interpreted with caution considering several limitations. First, this study has focused on peripheral immune cell and neopterin levels, making it possible that outcomes differ from measurements in the brain or in the CSF. Second, high levels of between-study heterogeneity were recorded for most several immune/inflammatory markers (i.e., B cells, Tc cells, Th cells, Th17 cells, Treg cells, NLR) assessed in our analysis. While the meta-analytic approach attempts to adjust for methodological confounders, the procedure was limited by the number of available studies and the information that could be extracted from the included studies. Unexplained heterogeneity could be related to potential moderators that were not consistently addressed in the different studies, such as BMI [59] and/or the presence of comorbid psychiatric conditions [60]. BMI was recorded in only four studies [29,32,37,40]; the absence of comorbid psychiatric comorbid conditions was specified in only eight studies [31,32,35,36,38,40,41,46] e. The presence or absence of comorbid immune-mediated/inflammatory conditions and/or of signs of immune activation at the time of blood withdrawal could also have influenced our findings. Fortunately, the absence of these conditions was specified in, in total, nineteen of included studies (i.e., 76 % of publications included) [22,23,25,27,29,30,31,32,34,35,36,37,38,39,40,41,44,45,46]. Therefore, we consider that our findings might have been only slightly influenced by this moderator. Another important limitation is the heterogeneity of the disorder. In a total of nine publications, individuals were diagnosed with an autistic disorder [22,23,24,26,27,28,30,36,37], in eleven, with an ASD [31,33,34,35,40,41,42,43,44,45,46]) in one, with regressive autism [29], and in three, with several autistic conditions, such as autistic disorder, Asperger’s syndrome, and/or pervasive developmental disorder, not otherwise specified [32,38,39]. Different periods of time and advancements in ASD diagnostic capability and criteria make it difficult to compare results from early and current studies. However, we attempted to control for these aspects by including year of publication in our meta-regression analyses and by carrying out subgroup meta-analyses based on the diagnostic criteria applied. Unfortunately, another limitation of our study was the small number of studies that could be included in the meta-regression and subgroup analyses for some immune/inflammatory markers. Finally, causal associations between the immune/inflammatory markers assessed and ASD could not be established due to a lack of longitudinal and interventional studies in the present meta-analysis.

## 5. Conclusions

Taken together, findings from this meta-analysis suggest that at least a subgroup of subjects with ASD might show abnormal levels of immune cells (i.e., increased WBC count, elevated peripheral levels of monocytes, neutrophils, Tc cells, NLR) and increased peripheral levels of the immune cell activation marker neopterin, compared to TD controls. These findings suggest the existence of a (chronic) activated IRS in ASD. In particular, individuals with ASD might be characterized by a chronic activation of the monocyte/macrophage system, and by abnormal blood levels of different lymphocyte subpopulations. Stratification of individuals with ASD based on (immune/inflammatory) parameters could be a method for improving diagnosis and increasing the effectiveness of interventions (personalized medicine). However, rigorously designed and larger longitudinal studies, including more homogeneous samples and recording potential moderators such medication status, BMI, and/or the presence of comorbid mental and/or medical disorders are urgently needed to confirm our findings. 

## Figures and Tables

**Table 1:** Characteristics of included studies.

Name of First Autor (Date of Publication)	Country	Diagnostic Criteria (Diagnose)	(S)S Interview (Name)	*N* (ASD, TD)	Mean Age (SD)	% of Males	Medication
Sweeten et al. (2003) [22]	USA	DSM-IV (autistic disorder)	ADI-R	ASD (*n* = 31) TD (*n* = 28)	6.0 (2.80) 6.5 (2.50)	87% 86%	No No
Enstrom et al. (2009) [23]	USA	DSM-IV (autistic disorder)	ADI-R ADOS	ASD (*n* = 17) TD (*n* = 16)	3.9 (0.7) 3.3 (0.62)	82% 81%	NG NG
Onore et al. (2009) [24]	USA	DSM-IV (autistic disorder)	ADI-R ADOS	ASD (*n* = 34) TD (*n* = 26)	3.83 (0.27) 3.71 (0.37)	85% 81%	NG NG
Saresella et al. (2009) [25]	Italy	DSM-IV (autistic disorder)	NG	ASD (*n* = 29) TD (*n* = 20)	13 (3.00) 11 (3.00)	70% 55%	No No
Mostafa et al. (2010) [26]	Egypt	DSM-IV (autistic disorder)	NG	ASD (*n* = 30) TD (*n* = 30)	8.27 (2.66) 8.03 (2.50)	73% 73%	NG NG
Ashwood et al. (2011) [27]	USA	DSM-IV (autistic disorder)	ADI-R ADOS	ASD (*n* = 70) TD (*n* = 35)	3.8 (NG) 3.5 (NG)	83% 83%	NG NG
Heuer et al. (2012) [28]	USA	DSM-IV (autistic disorder)	ADI-R ADOS	ASD (*n* = 42) TD (*n* = 31)	6.83 (NG) 6.00 (NG)	88% 77%	NG NG
Wasilewska et al. (2012) [29]	Poland	DSM-IV/ICD-10 (regressive autism)	NG	ASD (*n* = 24) TD (*n* = 24)	4.25 (1.70) 4.25 (2.20)	96% 96%	No No
Bodur et al. (2014) [30]	Turkey	DSM-IV (autistic disorder)	NG	ASD (*n* = 23) TD (*n* = 21)	5.9 (2.6) 5.8 (2.3)	96% 81%	NG NG
Tonhajzerova et al. (2015) [31]	Slovakia	DSM-5 (ASD, no-regression)	NG	ASD (*n* = 15) TD (*n* = 20)	9.3 (0.7) 9.6 (0.8)	87% sex-matched	No No
Zhao et al. (2015) [32]	China	DSM-IV *n* = 75 autistic disorder, *n* = 3 Asperger’s *n* = 2 PDD-NOS	NG	ASD (*n* = 80) TD (*n* = 80)	3.69 (1.30) 3.69 (1.30)	80% 80%	No No
Ahmad et al. (2016) [33]	KSA	DSM-5 (ASD)	NG	ASD (*n* = 40) TD (*n* = 32)	7.69 (2.26) 7.76 (2.45)	75% 75%	No No
Lopez-Cacho et al. (2016) [34]	Spain	DSM-5 (ASD)	NG	ASD (*n* = 59) TD (*n* = 26)	24.44 (6.24) 30.69 (6.28)	73% 27%	NG NG
Siniscalco et al. (2016) [35]	Italy	DSM-5 (ASD; No Asperger’s)	ADOS	ASD (*n* = 104) TD (*n* = 31)	6.7 (3.6) 5.2 (3.4)	75% 61%	No No
Ashaat et al. (2017) [36]	Egypt	ICD-10 (autistic disorder)	NG	ASD (*n* = 60) TD (*n* = 60)	8.7 (1.3) 7.9 (1.6)	93% 77%	NG NG
Pardo et al. (2017) [37]	USA	DSM-IV-TR (autistic disorder)	ADI-R, ADOS	ASD (*n* = 104) TD (*n* = 54)	4.41 (1.27) 3.64 (1.11)	83% 76%	NG
Kutlu and Cehver (2018) [38]	Turkey	ICD-10 *n* = 11 autistic disorder *n* = 53 PDD-NOS	NG	ASD (*n* = 64) TD (*n* = 64)	3.43 (1.03) age-matched	81% sex-matched	No No
Hesapcioglu et al. (2019) [39]	Turkey	DSM-IV/ICD-10 *n* = 30 autistic disorder *n* = 15 PDD-NOS	NG	ASD (*n* = 45) TD (*n* = 43)	13.51 (4.21) 11.90 (3.73)	80% 77%	No NG
Moaaz et al. (2019) [40]	Egypt	DSM-5 (ASD)	NG	ASD (*n* = 44) TD (*n* = 45)	7.2 (2.2) 7.1 (2.1)	79% sex-matched	NG NG
Abd-Allah et al. (2020) [41]	Egypt	DSM-5 (ASD)	NG	ASD (*n* = 35) TD (*n* = 35)	4.7 (2.4) 5.4 (2.4)	69% 54%	NG NG
Alpay et al. (2020) [42]	Turkey	NG (ASD)	NG	ASD (*n* = 30) TD (*n* = 30)	children	NG	NG NG
Rose et al. (2020) [43]	USA	DSM-IV (ASD)	ADI-R ADOS	ASD (*n* = 10) TD (*n* = 15)	children	sex-matched	No No
Ceylan et al. (2021) [44]	Turkey	DSM-5 (ASD)	NG	ASD (*n* = 48) TD (*n* = 38)	9.4 (4.1) 9.8 (4.1)	77% 60%	Yes (*n* = 27) NG
De Giacomo et al. (2021) [45]	Italy	DSM-5 (ASD)	ADOS	ASD (*n* = 26) TD (*n* = 16)	8.3 (3.6) 9.9 (5.7)	81% 81%	No No
Topal et al. (2021) [46]	Turkey	DSM-5 (ASD)	K-SADS-PL (6–18 years) No (<6 years)	ASD (*n* = 72) TD (*n* = 70)	8.3 (3.2) 8.4 (3.8)	76% 70%	No No

**Table 2:** Meta-analyses of cross-sectional comparisons of immune/inflammatory markers between individuals with ASD and TD controls.

Marker	k	*N* ASD	*N* TD	Effect Size (ASD vs. TD)			Heterogeneity		
				Hedge’s *g* (95% CI)	*Z* Value	*p*-Value	*Q*	*p*-Value	*I* ^2^
WBC	10	526	436	0.286 (0.125–0.448)	3.477	**0.001**	13.644	0.136	34.04%
Neutrophils	6	314	270	0.287 (0.085–0.488)	2,784	**0.005**	7.272	0.201	31.25%
Monocytes	11	629	488	0.355 (0.173–0.536)	3.825	**<0.001**	21.696	0.017	53.91%
Lymphocytes	6	374	329	−0.055 (−0.229–0.119)	−0.624	0.533	6.599	0.252	24.23%
B cells	5	215	136	−0.090 (−0.583–0.404)	−0.356	0.722	19.816	0.001	79.81%
NK cells	7	320	168	0.364 (0.021–0.706)	2.083	**0.037**	17.998	0.006	66.66%
T cells	5	215	136	0.024 (−0.367–0.414)	0.119	0.905	12.491	0.014	67.98%
Tc cells	4	173	105	0.726 (0.111–1.342)	2.313	**0.021**	16.692	0.001	82.03%
Th cells	5	233	165	−0.346 (−1.102–0.410)	−0.896	0.370	49.481	<0.001	91.92%
Th/Tc cells ratio	3	103	70	−0.419 (−0.728–−0.110)	−2.661	**0.008**	0.923	0.630	0.00%
Tregs	7	233	188	−1.304 (−2.611–0.004)	−1.954	0.051	183.821	<0.001	96.74%
Th17	3	102	93	2.058 (−0.250–4.366)	1.747	0.081	75.642	<0.001	97.36%
NLR	4	181	177	0.686 (0.033–1.339)	2.058	**0.040**	30.834	<0.001	90.27%
Neopterin	3	134	129	1.159 (0.624–1.693)	4.246	**<0.001**	7.095	0.029	71.809

WBC: Publication bias markers: Orwin’s FSN = 5, Egger’s regression test, *p*-value (2-tailed) = 0.167. Monocytes: Publication bias markers: Orwin’s FSN = 8, Egger’s regression test, *p*-value (2-tailed) = 0.099 (Supplementary Figure S1).

## Supplementary Materials

The following supporting information can be downloaded at: https://www.mdpi.com/article/10.3390/ijms232214329/s1.
