# Supplementary material for: Activation of the Monocyte/Macrophage System and Abnormal Blood Levels of Lymphocyte Subpopulations in Individuals with Autism Spectrum Disorder: A Systematic Review and Meta-Analysis
Source: Int J Mol Sci. 2022 Nov 18;23(22):14329. doi: 10.3390/ijms232214329 (PMC9699353; doi:10.3390/ijms232214329)
Supplement: Supplementary file 1 [file ijms-23-14329-s001.zip › ijms-1941773-SI.pdf]

Supplementary Table S1. The PRISMA checklist

Section and Topic	Item #	Checklist item	Location where item is reported
**TITLE**			
Title	1	Identify the report as a systematic review.	1
**ABSTRACT**			
Abstract	2	See the PRISMA 2020 for Abstracts checklist.	2
**INTRODUCTION**			
Rationale	3	Describe the rationale for the review in the context of existing knowledge.	3
Objectives	4	Provide an explicit statement of the objective(s) or question(s) the review addresses.	3
**METHODS**			
Eligibility criteria	5	Specify the inclusion and exclusion criteria for the review and how studies were grouped for the syntheses.	4
Information sources	6	Specify all databases, registers, websites, organisations, reference lists and other sources searched or consulted to identify studies. Specify the date when each source was last searched or consulted.	4
Search strategy	7	Present the full search strategies for all databases, registers and websites, including any filters and limits used.	4
Selection process	8	Specify the methods used to decide whether a study met the inclusion criteria of the review, including how many reviewers screened each record and each report retrieved, whether they worked independently, and if applicable, details of automation tools used in the process.	4
Data collection process	9	Specify the methods used to collect data from reports, including how many reviewers collected data from each report, whether they worked independently, any processes for obtaining or confirming data from study investigators, and if applicable, details of automation tools used in the process.	4
Data items	10a	List and define all outcomes for which data were sought. Specify whether all results that were compatible with each outcome domain in each study were sought	4

Section and Topic	Item #	Checklist item	Location where item is reported
		(e.g. for all measures, time points, analyses), and if not, the methods used to decide which results to collect.	
	10b	List and define all other variables for which data were sought (e.g. participant and intervention characteristics, funding sources). Describe any assumptions made about any missing or unclear information.	4
Study risk of bias assessment	11	Specify the methods used to assess risk of bias in the included studies, including details of the tool(s) used, how many reviewers assessed each study and whether they worked independently, and if applicable, details of automation tools used in the process.	4 - 5
Effect measures	12	Specify for each outcome the effect measure(s) (e.g. risk ratio, mean difference) used in the synthesis or presentation of results.	5-6
Synthesis methods	13a	Describe the processes used to decide which studies were eligible for each synthesis (e.g. tabulating the study intervention characteristics and comparing against the planned groups for each synthesis (item #5)).	5-6
	13b	Describe any methods required to prepare the data for presentation or synthesis, such as handling of missing summary statistics, or data conversions.	5-6
	13c	Describe any methods used to tabulate or visually display results of individual studies and syntheses.	5-6
	13d	Describe any methods used to synthesize results and provide a rationale for the choice(s). If meta-analysis was performed, describe the model(s), method(s) to identify the presence and extent of statistical heterogeneity, and software package(s) used.	5-6
	13e	Describe any methods used to explore possible causes of heterogeneity among study results (e.g. subgroup analysis, meta-regression).	5-6
	13f	Describe any sensitivity analyses conducted to assess robustness of the synthesized results.	5
Reporting bias assessment	14	Describe any methods used to assess risk of bias due to missing results in a synthesis (arising from reporting biases).	5
Certainty assessment	15	Describe any methods used to assess certainty (or confidence) in the body of evidence for an outcome.	5
**RESULTS**			
Study selection	16a	Describe the results of the search and selection process, from the number of records identified in the search to the number of studies included in the review,	7

Section and Topic	Item #	Checklist item	Location where item is reported
		ideally using a flow diagram.	
	16b	Cite studies that might appear to meet the inclusion criteria, but which were excluded, and explain why they were excluded.	7
Study characteristics	17	Cite each included study and present its characteristics.	7
Risk of bias in studies	18	Present assessments of risk of bias for each included study.	7-13
Results of individual studies	19	For all outcomes, present, for each study: (a) summary statistics for each group (where appropriate) and (b) an effect estimate and its precision (e.g. confidence/credible interval), ideally using structured tables or plots.	7-13
Results of syntheses	20a	For each synthesis, briefly summarise the characteristics and risk of bias among contributing studies.	7-13
	20b	Present results of all statistical syntheses conducted. If meta-analysis was done, present for each the summary estimate and its precision (e.g. confidence/credible interval) and measures of statistical heterogeneity. If comparing groups, describe the direction of the effect.	7-13
	20c	Present results of all investigations of possible causes of heterogeneity among study results.	7-13
	20d	Present results of all sensitivity analyses conducted to assess the robustness of the synthesized results.	7-13
Reporting biases	21	Present assessments of risk of bias due to missing results (arising from reporting biases) for each synthesis assessed.	7-13
Certainty of evidence	22	Present assessments of certainty (or confidence) in the body of evidence for each outcome assessed.	7-13
**DISCUSSION**			
Discussion	23a	Provide a general interpretation of the results in the context of other evidence.	14
	23b	Discuss any limitations of the evidence included in the review.	15-16
	23c	Discuss any limitations of the review processes used.	15-16

Section and Topic	Item #	Checklist item	Location where item is reported
	23d	Discuss implications of the results for practice, policy, and future research.	15-16
**OTHER INFORMATION**			
Registration and protocol	24a	Provide registration information for the review, including register name and registration number, or state that the review was not registered.	2,4
	24b	Indicate where the review protocol can be accessed, or state that a protocol was not prepared.	2,4
	24c	Describe and explain any amendments to information provided at registration or in the protocol.	
Support	25	Describe sources of financial or non-financial support for the review, and the role of the funders or sponsors in the review.	16
Competing interests	26	Declare any competing interests of review authors.	16
Availability of data, code and other materials	27	Report which of the following are publicly available and where they can be found: template data collection forms; data extracted from included studies; data used for all analyses; analytic code; any other materials used in the review.	9

Supplementary Table S2: Quality assessment of included studies

Study	S.1	S.2	S.3	S.4	C.1	E.1	E.2	E.3	NOS total score
Sweeten et al. (2003)	*	*	*	-	**	*	-	-	6
Enstrom et al. (2009)	*	*	*	*	**	*	*	-	8
Onore et al. (2009)	*	*	*	*	**	*	*	-	8
Saresella et al. (2009)	-	-	-	-	**	*	-	-	3
Mostafa et al. (2010)	-	*	*	*	**	*	-	-	6
Ashwood et al. (2011)	*	*	*	*	**	*	*	-	8
Heuer et al. (2012)	*	*	*	*	**	*	*	-	8
Wasilewska et al. (2012)	*	*	*	-	**	*	-	-	6
Bodur et al. (2014)	-	*	*	*	**	*	*	*	8

Tonhajzerova et al. (2015)	*	-	-	*	**	*	-	-	5
Zhao et al. (2015)	*	-	-	*	**	*	*	-	6
Ahmad et al. (2016)	-	*	*	-	**	*	-	-	5
Lopez-Cacho et al (2016)	-	*	*	-	**	*	-	-	5
Siniscalco et al. (2016)	-	*	*	-	**	*	*	-	6
Ashaat et al. (2017)	*	*	*	-	**	*	-	-	6
Pardo et al. (2017)	*	*	*	-	**	*	-	-	6
Kutlu and Cehver (2018)	-	*	-	-	**	*	-	*	5
Hesapcioglu et al. (2019)	*	*	-	-	**	*	-	*	6
Moaaz et al. (2019)	-	*	*	-	**	*	-	-	5
Abd-Allah et al. (2020)	-	*	*	-	**	*	*	-	6
Alpay et al. (2020)	-	*	-	*	-	-	-	-	2
Rose et al. (2020)	*	*	*	*	**	*	*	-	8
Ceylan et al. (2021)	-	*	*	*	**	*	*	*	8
De Giacomo et al. (2021)	-	*	*	-	**	*	*	-	6
Topal et al. (2021)	-	*	*	*	**	*	*	-	7

Supplementary Table S3. Immune/inflammatory markers assessed

Name of first autor (date of publication)	WBC	Neutrophils	Monocytes	Lymphocytes	B	NK	T	Tc	Th	Th/Tc	Treg	Th17	NLR	Neopterin
Sweeten et al. (2003)			+											+
Enstrom et al. (2009=						+								

Onore et al. (2009)												+		
Saresella et al. (2009)					+	+	+	+	+	+				
Mostafa et al. (2010)											+			
Ashwood et al. (2011)	+	+	+		+	+	+	+	+					
Heuer et al. (2012)			+		+		+							
Wasilewska et al. (2012)	+				+	+	+	+	+	+				
Bodur et al. (2014)														+
Tonhajzerova et al. (2015)	+	+	+	+										
Zhao et al. (2015)	+		+	+										+
Ahmad et al. (2016)											+	+		
López-Cacho et al. (2016)			+		+	+	+	+	+	+	+			
Siniscalco et al. (2016)						+								
Ashaat et al. (2017)									+					
Pardo et al. (2017)	+		+	+										
Kutlu and Cevher (2018)	+	+	+	+									+	
Hesapcioglu et al. (2019)	+	+	+	+									+	
Moaaz et al. (2019)	+										+	+		
Abd-Allah et al. (2020)	+													
Rose et al. (2020)											+	)		
Alpay et al. (2020)													+	
Ceylan et al. (2021)	+	+	+											
De Giacomo et al. (2021)						+					+			
Topal et al. (2021)		+	+	+									+	

Supplementary Table S4: Results of the leave-one-out analysis

Marker	Excluded study	*g*	95% CI	*p*	I^2
WBC	Ashwood et al. (2011)	0.285	0.105-0.471	**0.002**	40.99%
	Wasilewska et al. (2012)	0.270	0.102-0.444	**0.002**	37.33%
	Tonhajzerova et al. (2015)	0.270	0.105-0.441	**0.002**	36.68%
	Zhao et al. (2015)	0.331	0.007-0.170	**0.000**	22.26%
	Pardo et al. (2017)	0.261	0.086-0.441	**0.004**	34.42%
	Kutlu and Cevher (2018)	0.320	0.148-0.498	**0.000**	33.18%
	Hesapcioglu et al. (2019)	0.314	0.143-0.491	**0.000**	35.52%
	Moaaz et al. (2019)	0.238	0.087-0.394	**0.002**	18.63%
	Abd-Allah et al. (2020)	0.311	0.140-0.488	**0.000**	36.59%
	Ceylan et al. (2021)	0.269	0.095-0.448	**0.003**	37.43%
Neutrophils	Ashwood et al. (2011)	0.348	0.14-0.549	**0.001**	17.50%
	Tonhajzerova et al. (2015)	0.278	0.048-0.508	**0.018**	44.75%
	Kutlu and Cevher (2018)	0.288	0.031-0.546	**0.028**	44.73%
	Hesapcioglu et al. (2019)	0.340	0.130-0.550	**0.001**	24.42%
	Ceylan et al. (2021)	0.254	0.018-0.490	**0.035**	40.18%
	Topal et al. (2021)	0.200	0.011-0.388	**0.038**	0.00%

Monocytes	Sweeten et al. (2003)	0.344	0.150-0.538	**0.001**	57.43%
	Ashwood et al. (2011)	0.388	0.197-0.579	**0.000**	53.91%
	Heuer et al. (2012)	0.376	0.180-0.572	**0.000**	57.19%
	Tonhajzerova et al. (2015)	0.307	0.147-0.466	**0.000**	39.67%
	Zhao et al. (2015)	0.387	0.189-0.585	**0.000**	54.27%
	López-Cacho et al. (2016)	0.382	0.189-0.575	**0.000**	55.85%
	Pardo et al. (2017)	0.345	0.143-0.547	**0.001**	57.03%
	Kutlu and Cevher (2018)	0.391	0.199-0.582	**0.000**	52.46%
	Hesapcioglu et al. (2019)	0.323	0.135-0.510	**0.001**	52.52%
	Ceylan et al. (2020)	0.316	0.134-0.498	**0.001**	50.26%
	Topal et al. (2021)	0.355	0.150-0.561	**0.001**	58.24%
Lymphocytes	Tonhajzerova et al. (2015)	-0.085	-0.249-0.080	0.313	13.44%
	Zhao et al. (2015)	-0.075	-0.292-0.142	0.497	35.11%
	Pardo et al. (2017)	-0.044	-0.264-0.176	0.695	39.13%
	Kutlu and Cevher (2018)	0.019	-0.146-0.184	0.820	0.00%
	Hesapcioglu et al. (2019)	-0.049	-0.259-0.162	0.649	39.35%
	Topal et al. (2021)	-0.082	-0.291-0.126	0.439	32.37%
B cells	Saresella et al. (2009)	0.058	-0.440-0.556	0.819	77.73%

	Ashwood et al. (2011)	-0.238	0.316- -0.841	0.400	77.63%
	Heuer et al. (2012)	-0.013	0.573- -0.045	0.964	81.39%
	Wasilewska et al. (2012)	-0.019	0.562- -0.063	0.950	82.78%
	López-Cacho et al. (2016)	-0.248	0.294- -0.897	0.370	78.12%
NK cells	Enstrom et al. (2009)	0.334	-0.051-0.720	0.089	71.61%
	Saresella et al. (2009)	0.416	0.037-0.796	0.032	69.75%
	Ashwood et al. (2011)	0.340	-0.080-0.760	0.113	71.63%
	Wasilewska et al. (2012)	0.243	-0.029-0.516	0.080	41.69%
	López-Cacho et al. (2016)	0.405	0.004-0.806	0.048	70.24%
	Siniscalso et al. (2016)	0.343	-0.079-0.765	0.111	71.76%
	De Giacomo et al. (2021)	0.474	0.165-0.783	0.003	54.53%
T cells	Saresella et al. (2009)	-0.072	-0.507-0.364	0.747	70.84%
	Ashwood et al. (2011)	-0.030	-0.545-0.485	0.910	74.24%
	Heuer et al. (2012)	-0.061	-0.533-0.411	0.799	71.39%
	Wasilewska et al. (2012)	0.182	-0.086-0.449	0.184	21.83%
	López-Cacho et al. (2016)	0.077	-0.412-0.567	0.757	73.31%
Tc cells	Saresella et al. (2009)	0.812	-0.014-1.637	0.054	87.89%
	Ashwood et al. (2011)	0.962	0.373-1.552	**0.001**	68.31%

	Wasilewska et al. (2012)	0.456	-0.023-0.935	0.062	64.99%
	López-Cacho et al. (2016)	0.698	-0.178-1.575	0.118	86.95%
Th cells	Saresella et al. (2009)	-0.574	-1.396-0.249	0.171	92.33%
	Ashwood et al. (2011)	-0.489	-1.417-0.438	0.301	92.48%
	Wasilewska et al. (2012)	-0.044	-0.781-0.693	0.906	90.58%
	López-Cacho et al. (2016)	-0.467	-1.410-0.477	0.332	93.24%
	Ashaat et al. (2017)	-0.158	-0.979-0.662	0.705	90.34%
Th/Tc cells ratio	Saresella et al. (2009)	-0.529	-0.888--0.170	0.004	
	Wasilewska et al. (2012)	-0.390	-0.759--0.021	0.038	
	López-Cacho et al. (2016)	-0.366	-0.781--0.126	0.084	
Th17 cells	Onore et al. (2009)	3.066	0.126-6.007	**0.041**	97.39%
	Ahmad et al. (2016)	2.308	-2.136-6.753	0.309	98.66%
	Moaaz et al. (2019)	0.827	-0.677-2.330	0.281	92.15%
Treg cells	Mostafa et al. (2010)	-1.293	-2.870-0.284	0.108	97.25%
	Wasilewska et al. (2012)	-1.758	-2.987- -0.529	**0.005**	95.66%
	Ahmad et al. (2016)	-0.875	-2.123-0.373	0.169	96.07%
	López-Cacho et al. (2016)	-1.498	-3.067-0.071	0.061	96.98%
	Moaaz et al. (2020)	-0.921	-2.161-0.319	0.145	95.83%

	Rose et al. (2020)	-1.357	-2.850-0.135	0.075	97.28%
	De Giacomo et al. (2021)	-1.425	-2.968-0.118	0.070	97.25%
NLR	Kutlu and Cevher (2018)	0.851	-0.101-1.803	0.080	92.89%
	Hesapcioglu et al. (2019)	0.919	0.096-1.742	**0.029**	91.71%
	Alpay et al. (2020)	0.310	-0.009-0-628	0.057	56.61%
	Topal et al. (2021)	0.742	-0.259-1.742	0.146	93.43%
Neopterin	Sweeten et al. (2003)	1.161	0.281-2.042	**0.010**	84.52%
	Bodur et al. (2014)	1.380	0.913-1.848	**<0.001**	53.81%
	Zhao et al. (2015)	0.905	0.500-1.310	**<0.001**	1.88%

Supplementary Table S5.

Outcome	Moderator variable	*k*	Coefficient (95% CI)	*Z*-value	*p*-value
WBC	Year of publication	10	-0.007 (-0.063-0.0498)	-0.23	0.817
	Mean difference in age	10	-0.087 (-0.441-0.267)	-0.48	0.630
	Mean difference in % of males	10	0.001 (-0.027-0.029)	0.08	0.938
Neutrophils	Year of publication	6	0.045 (-0.001-0.093)	1.93	0.053
	Mean difference in age	6	-0.262 (-0.585-0.060)	-1.59	0.111
	Mean difference in % of males	6	0.020 (-0.012-0.053)	1.22	0.222
Monocytes	Year of publication	10	0.002 (-0.068-0.072)	0.06	0.949
	Mean difference in age	10	-0.071 (-0.268-0.125)	-0.71	0.476
	Mean difference in % of males	10	-0.011 (-0.037-0.015)	-0.81	0.419
Lymphocytes	Year of publication	6	-0.110 (-0.287-0.067)	-1.21	0.225
	Mean difference in age	6	-0.062 (-0.703-0.578)	-0.19	0.848
	Mean difference in % of males	6	-0.042 (-0.172-0.088)	-0.640	0.522
NK cells	Year of publication	7	-0.059 (-0.143-0.023)	-1.41	0.157
	Mean difference in age	7	-0.108 (-0.282-0.0669	-1.22	0.223

	Mean difference in % of males	7	-0.008 (-0.030-0.015)	-0.68	0.494
T reg cells	Year of publication	7	-0.123 (-0.487-0.242)	-0.66	0.509
	Mean difference in age	6	0.271 (-0.465-1.007)	0.72	0.471
	Mean difference in % of males	6	0.031 (-0.067-0.129)	0.62	0.535

Supplementary Figures:

Supplementary Figure S1:

WBC:

Monocytes:

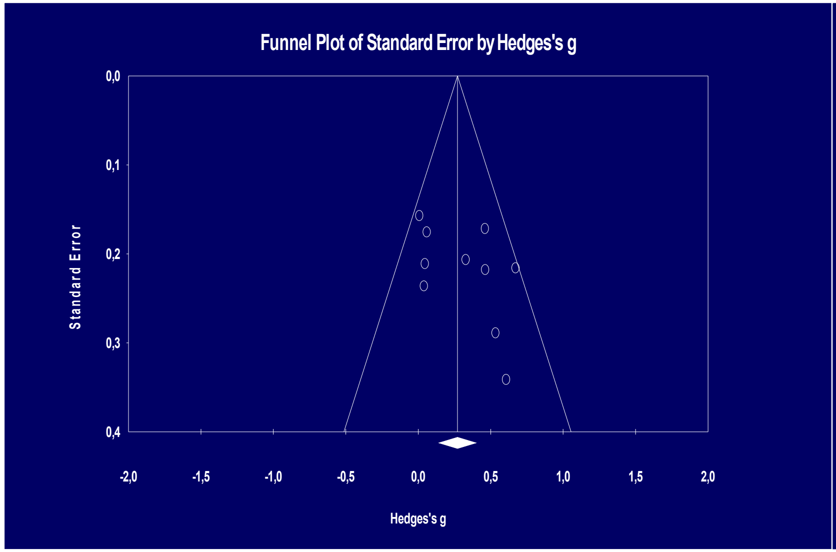
Funnel Plot of Standard Error by Hedges's g
Standard Error
0,0
0,1
0,2
0,3
0,4
-2,0
-1,5
-1,0
-0,5
0,0
0,5
1,0
1,5
2,0
Hedges's g

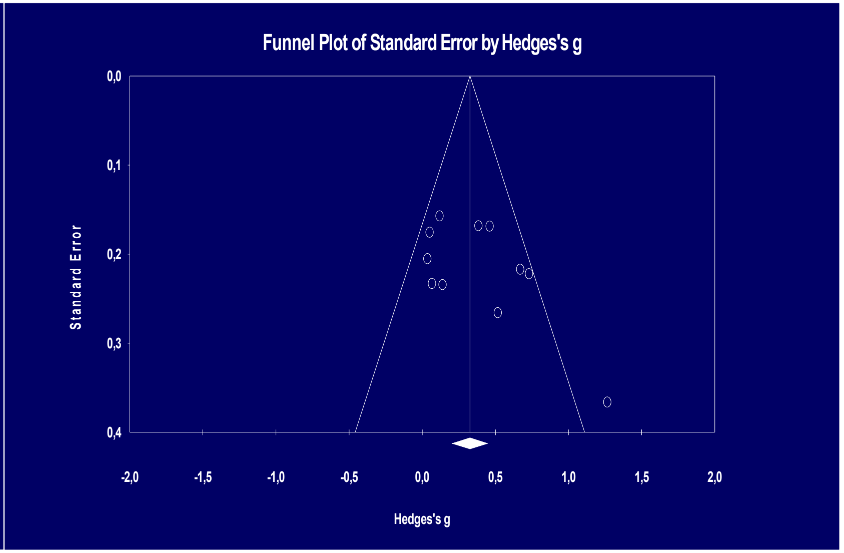
Funnel Plot of Standard Error by Hedges's g
Standard Error
0,0
0,1
0,2
0,3
0,4
-2,0
-1,5
-1,0
-0,5
0,0
0,5
1,0
1,5
2,0
Hedges's g
